# Supplementary figures and images for: The relative importance of DNA methylation and Dnmt2-mediated epigenetic regulation on Wolbachia densities and cytoplasmic incompatibility
Source: PeerJ. 2014 Dec 9;2:e678. doi: 10.7717/peerj.678 (PMC4266856; doi:10.7717/peerj.678)

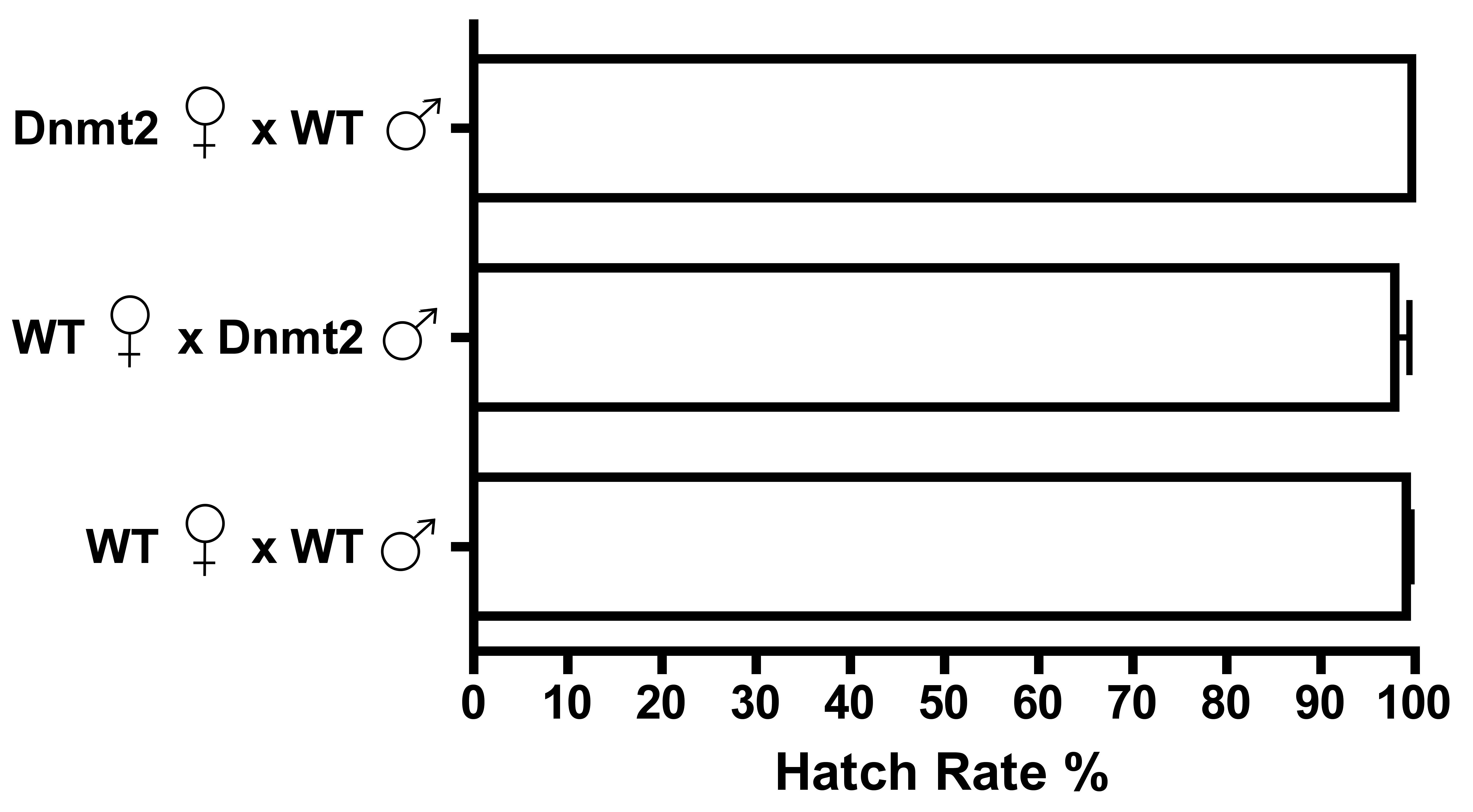

Supplement: Figure S1 — Overexpression of Dnmt2 by an Actin-Gal4 driver does not induce CI in uninfected males. Bars denote SEM. Dnmt2, overexpressing flies; WT, wild type flies. [file peerj-02-678-s001.png]

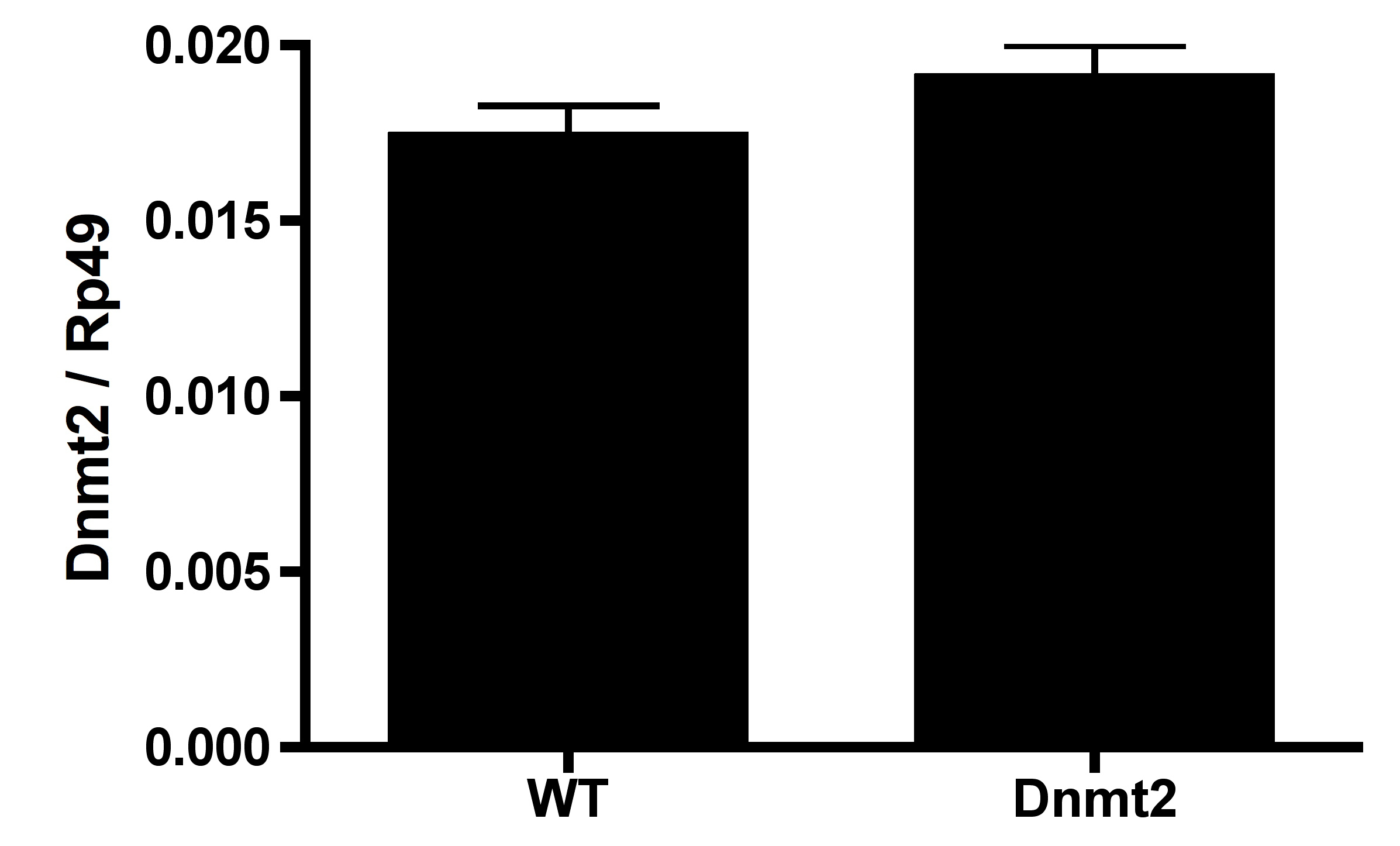

Supplement: Figure S2 — Dnmt2 is overexpressed 9.6% compared to wild type in testes using ananos-Gal4 driver. Bars denote SEM. WT, wild type; Dnmt2, Dnmt2 overexpressing. Rp49 is used as a control for gene expression. [file peerj-02-678-s002.png]

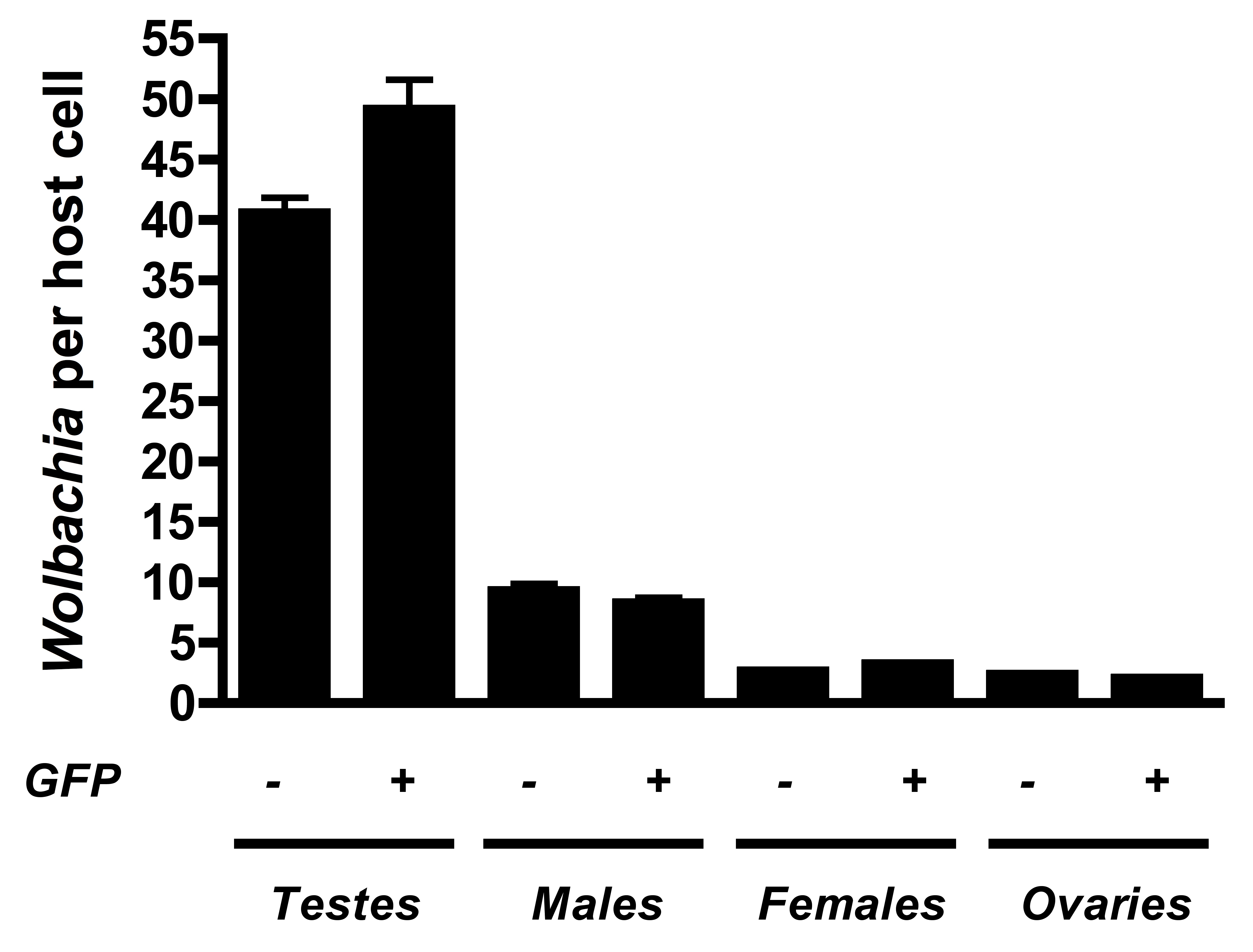

Supplement: Figure S3 — Expression of green fluorescent protein (GFP) does not reduce Wolbachia titers, as measured in whole males, females, testes, and ovaries. Wolbachia infection arises from the y1w∗ background. Bars denote SEM. ± indicates whether sample express GFP. [file peerj-02-678-s003.png]

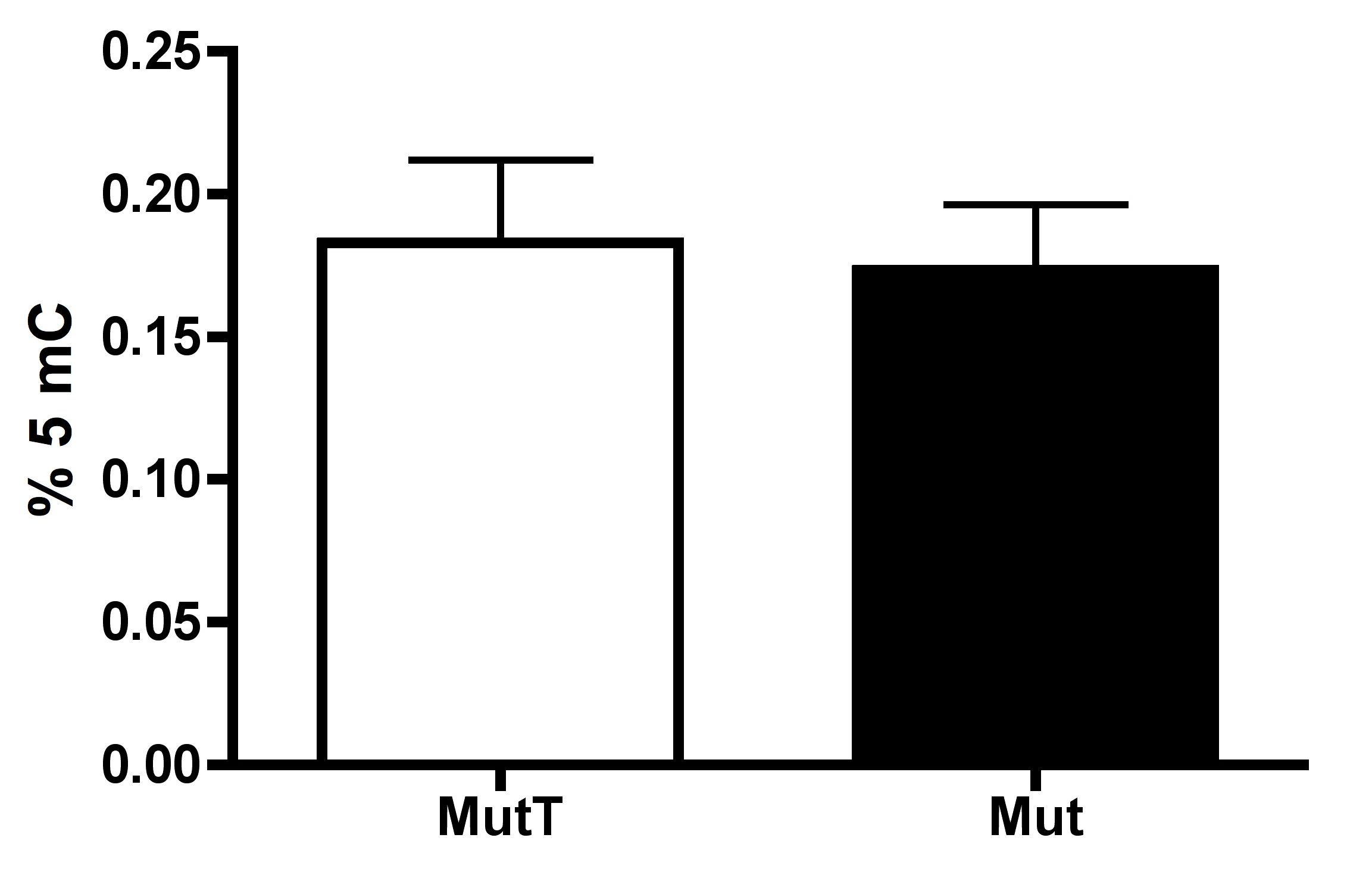

Supplement: Figure S4 — Testes from Drosophila melanogaster Dnmt2 mutants do not exhibit Wolbachia-induced increase in DNA methylation as measured by MethylFlash. Wolbachia infection arises from the W1118 background. Bars denote SEM. MutT, uninfected, Dnmt2 mutant; Mut, Wolbachia infected, Dnmt2 mutant. [file peerj-02-678-s004.png]

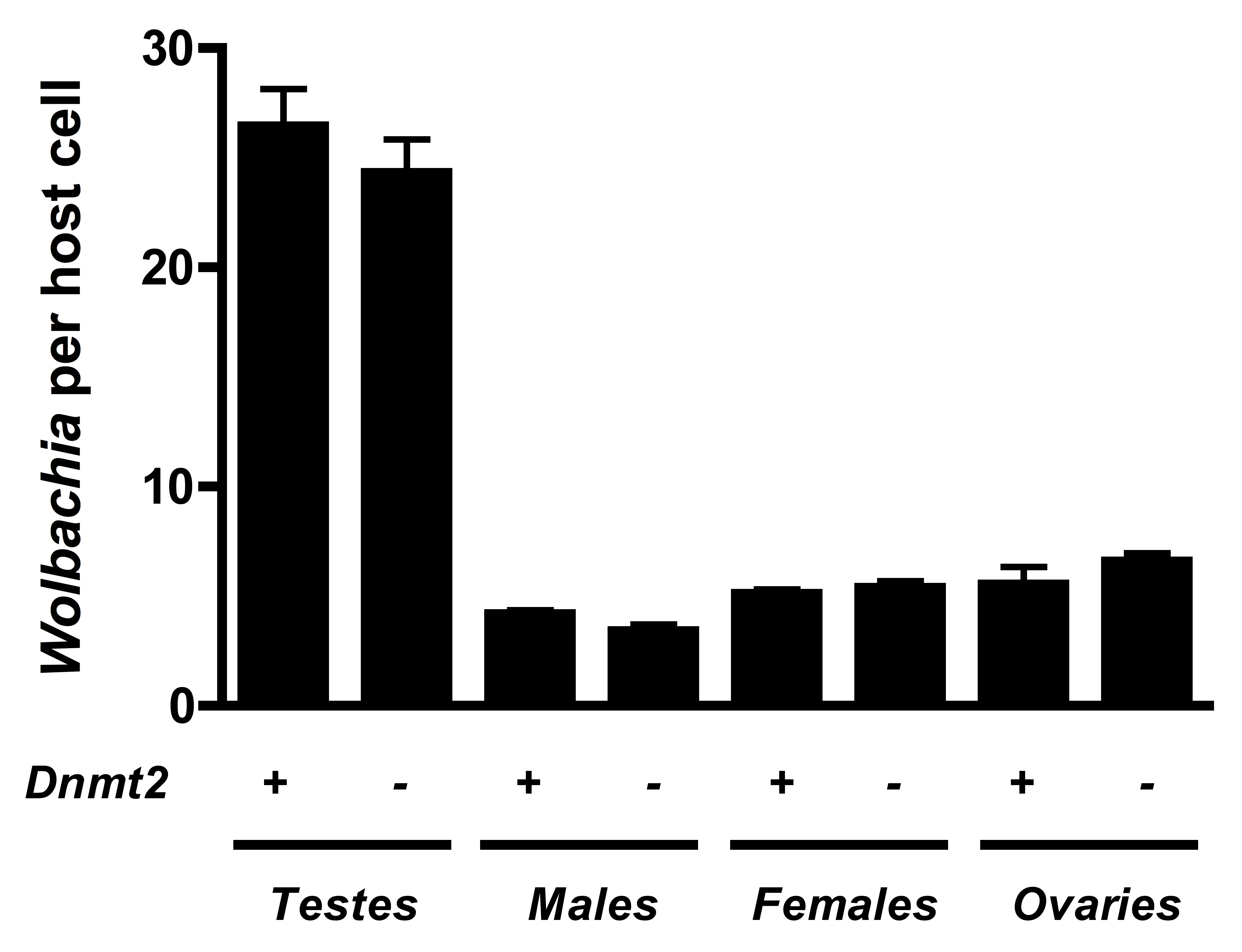

Supplement: Figure S5 — Loss of Dnmt2 does not affect Wolbachia titers in Drosophila melanogaster. Bars denote SEM. Dnmt2 +, wild type flies; Dnmt2 −, Dnmt2 mutant flies. [file peerj-02-678-s005.png]
